# Previously undiagnosed pulmonary arteriovenous malformation in hereditary hemorrhagic telangiectasia presenting as a brain abscess: a case report

**DOI:** 10.1186/s12883-026-05220-w

**Published:** 2026-08-11

**Authors:** Aisha Ghazanfar, Saba Zaidi, Shafiq Ur Rehman, Hazim Brohi

**Affiliations:** Department of Neuromedicine, Liaquat National Hospital, Karachi, Pakistan

**Keywords:** Brain abscess, Pulmonary arteriovenous malformation, Hereditary hemorrhagic telangiectasia, Paradoxical embolism, Transthoracic contrast echocardiography

## Abstract

**Introduction:**

Pulmonary arteriovenous malformations (PAVMs) are abnormal vascular connections that predispose to paradoxical embolism and brain abscess formation. They often remain undetected until severe complications occur, posing significant diagnostic challenges.

**Case presentation:**

We report the case of a young woman who presented with new onset focal seizures. Brain MRI with contrast revealed two ring enhancing lesions, suggestive of brain abscesses. The larger lesion was drained surgically, whereas the smaller one was conservatively treated with antibiotics. During her recovery, a sudden decline in her respiratory function was noted, which prompted additional investigations. CT pulmonary angiography showed pulmonary arteriovenous malformation; subsequently, she underwent a successful angioembolization. Based on the clinical presentation of this patient, the possibility of previously undiagnosed hereditary hemorrhagic telangiectasia (HHT) was considered.

**Conclusion:**

This case underscores the significance of evaluating PAVMs as a potential source of brain abscesses, particularly in young patients without apparent risk factors. Early recognition and management of PAVMs, including angioembolization, are essential to prevent recurrence and better outcomes.

**Supplementary Information:**

The online version contains supplementary material available at https://doi.org/10.1186/s12883-026-05220-w.

## Introduction

Pulmonary arteriovenous malformations (PAVMs) allow venous blood to bypass the pulmonary circulation, creating a right-to-left shunt that predisposes patients to paradoxical emboli and septic embolization. Although some patients show hypoxemia or dyspnea, the majority are asymptomatic, making diagnosis difficult. Brain abscess is a known complication occurring in 1–5% of PAVM patients [1].

PAVMs may occur sporadically or, more commonly, in association with hereditary hemorrhagic telangiectasia (HHT), an autosomal dominant vascular disorder caused by abnormal angiogenesis leading to telangiectasias and arteriovenous malformations involving mucocutaneous surfaces, visceral organs, and the central nervous system. HHT typically presents with recurrent epistaxis and may also be associated with gastrointestinal bleeding, hepatic involvement, and respiratory symptoms such as dyspnea due to PAVMs [2, 3].

Brain abscess remains an uncommon but recognized complication of PAVMs. However, diagnosis is often delayed when the underlying diagnosis of HHT has not yet been established clinically [3].

Here, we report a rare case of a 28-year-old woman who developed a brain abscess secondary to an undiagnosed PAVM, emphasizing the importance of early recognition of this potentially underdiagnosed but clinically serious condition.

## Case Presentation

A 28 year old right handed woman with no known comorbidities presented with a 15-day history of focal seizures involving her left arm. Four days before presentation, she developed a severe right-sided headache and one episode of vomiting, followed by sudden onset of left-sided weakness one day before arrival. She denied fever, trauma, loss of consciousness, or visual symptoms. Further history revealed recurrent spontaneous epistaxis and a family history of similar bleeding episodes in her mother and sisters, raising suspicion for hereditary hemorrhagic telangiectasia (HHT). There was no history of intravenous drug use. 

On examination, she was conscious and oriented, with focal left-sided weakness (MRC 3/5) in both upper and lower limbs and grade III digital clubbing. Breath sounds were clear.

Following suspicion of HHT, a targeted examination for mucocutaneous telangiectasias involving the oral mucosa, lips, tongue, fingertips, and skin was performed; however, no visible telangiectasias were identified. Oral examination did not show any significant dental disease or periodontal infection.

Laboratory studies revealed neutrophilic leukocytosis and an elevated hematocrit (Table 1). Bubble echocardiogram using standard agitated saline was negative. The study demonstrated no definitive appearance of microbubbles in the left-sided cardiac chambers within the expected cardiac cycle window, consistent with a Grade 0 right-to-left shunt on visual assessment.

**Table 1:** Patient laboratory results at admission and at day 7, with reference ranges

Laboratory workup	Values: Day 1	Values: Day 7	Normal Range
Hemoglobin	14.3 g/dl	15.2 g/dl	12-16 g/dl
Total leukocyte count	18.26 ×10⁹/L	19.26 ×10⁹/L	4–11 ×10⁹/L
Platelets	281 ×10⁹/L	346 ×10⁹/L	150–450 ×10⁹/L
INR	1.1	-	0.8–1.2
UREA	8.57 mmol/L	-	2.5–7.1 mmol/L
Creatinine	77.9 µmol/L	-	53–106 µmol/L
Troponin I	Negative	-	Negative
ESR	32 mm/hr	50 mm/hr	0–20 mm/hr
CRP	103 mg/L	-	0.0-5.0 mg/L
Urine Culture & Sensitivity	Negative	-	-
Fungal Culture & Sensitivity	Negative	-	-
Pus Culture & Sensitivity	Streptococcus milleri	-	-
CSF D/R			
CSF Glucose	2.72 mmol/L	7.49 mmol/L	2.8–4.4 mmol/L
CSF Protein	0.8 g/L	-	0.15–0.45 g/L
CSF WBC	12 cells/μL	Not repeated; differential: 85% polymorphs	0–5 cells/μL
CSF Culture & Sensitivity	Negative	-	-
CSF MTB PCR	Negative	-	-

*INR* International Normalized Ratio, *ESR* Erythrocyte Sedimentation Rate, *CRP* C-Reactive Protein, *HIV* Human Immunodeficiency Virus, *CSF* Cerebrospinal Fluid, *D/R* Detailed Report, *MTB* Mycobacterium tuberculosis

**Fig. 1:**
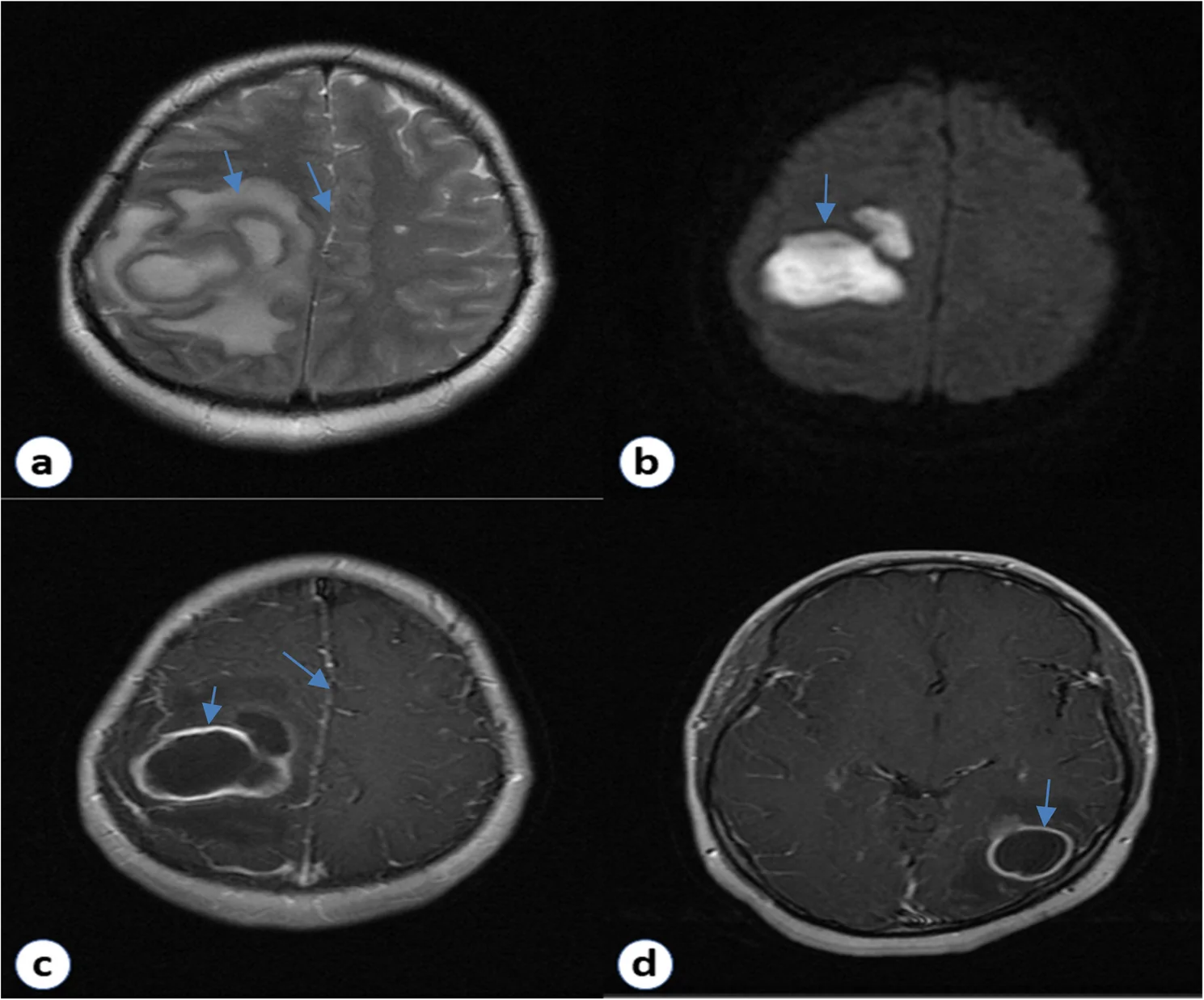
Brain MRI showing two ring-enhancing brain abscesses: **a** T2-weighted image demonstrating a right frontoparietal lesion with significant oedema and slight midline shift (arrows). **b** Diffusion-weighted image (DWI) showing restricted diffusion of the same ring-enhancing lesion, consistent with a brain abscess (arrow). **c** Brain magnetic resonance imaging showing a 2.9 × 4.6 × 3.0 cm ring-enhancing mass in the right superior frontoparietal region associated with significant oedema and mild midline shift (arrows). **d** MRI showing another ring-enhancing lesion in the posterolateral temporal region (3.2 × 2.6 cm) with mild diffuse meningeal enhancement (arrow).

A contrast enhanced MRI of the brain demonstrated two ring-enhancing lesions with restricted diffusion and surrounding oedema, consistent with brain abscesses (Figs. 1a–d).

She was started empirically on intravenous ceftriaxone and vancomycin. Metronidazole was not included initially because there was no clinical or radiological evidence of a sinus, dental, or otogenic source of infection. Seizures were managed with intravenous levetiracetam, with no further episodes during hospitalization.

Due to significant mass effect and oedema, neurosurgery performed a right image-guided frontal craniotomy with evacuation of the dominant abscess, draining approximately 10 mL of pus for culture. The second lesion was managed conservatively with antimicrobial therapy. Postoperatively, infectious disease specialists escalated antibiotics to meropenem and vancomycin while awaiting culture results.

Pus culture grew Streptococcus milleri and multidrug-resistant Acinetobacter. Although the Acinetobacter isolate was ultimately considered a likely procedural contaminant based on clinical correlation, its significance was initially uncertain. Following review by the infectious disease team, antimicrobial therapy was streamlined to ceftriaxone and trimethoprim/sulfamethoxazole. Blood, urine, and fungal cultures remained negative, and MTB DNA was not detected.

**Fig. 2:**
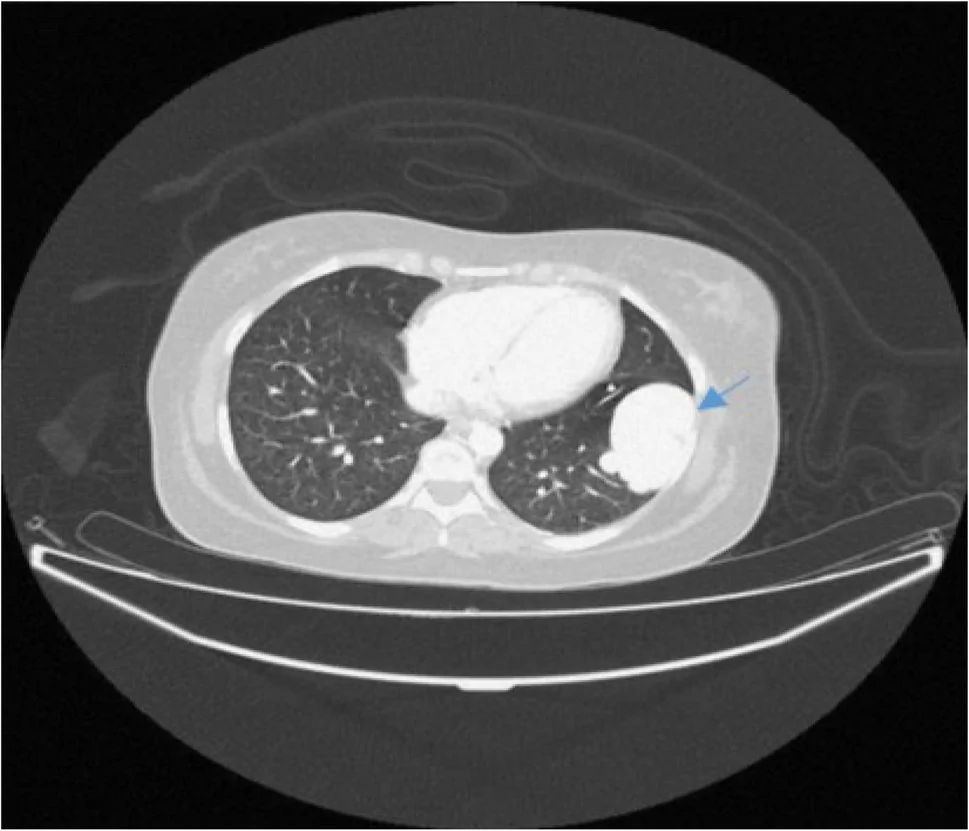
CT Pulmonary Angiogram showing a vascular anomaly within the lower lobe of the left lung, representing AV Malformation (arrow)

After two weeks, the patient returned with agitation, worsening headache, and vomiting. Given concern for persistent intracranial infection despite ongoing therapy, antimicrobial treatment was escalated to ertapenem with continuation of trimethoprim/sulfamethoxazole. That night, she developed hypoxia with oxygen saturation falling to 86% on room air, prompting transfer to a high-dependency unit. A repeat chest radiograph showed persistent haziness in the left lower zone but remained non-diagnostic. A CT pulmonary angiogram done revealed a vascular lesion within the left lower lobe, which enhanced in the arterial phase and drained into the left pulmonary vein, consistent with a PAVM (Fig. 2). No venous phase or three-dimensional reconstruction images were available for retrospective inclusion.

The next morning, her saturation dropped further to 78% despite high-flow oxygen, and she developed peripheral cyanosis requiring CPAP support. Cardiothoracic surgery recommended interventional management given her instability. The vascular and interventional radiology team performed coil embolization of the PAVM, which she tolerated well. Her oxygen requirement decreased to 2–3 L, and she was transferred back to the regular ward.

She was eventually discharged in stable condition with a plan for six weeks of intravenous administration of antibiotics. Based on the Curaçao criteria, she met three features, recurrent epistaxis, visceral AVM, and a positive family history, confirming a diagnosis of definite hereditary hemorrhagic telangiectasia [2].

## Discussion

This case emphasizes important diagnostic and management considerations in brain abscesses secondary to pulmonary arteriovenous malformations (PAVMs), particularly in young patients without an obvious primary infectious source. Brain abscess is a rare condition, with an incidence of approximately 0.3–1.3 cases per 100,000 population per year, accounting for nearly 8% of intracranial infections, yet it carries significant morbidity if not promptly recognized and treated [4].

Brain abscess symptoms depend on the localization of the lesion. Despite the fact that headache is the most common symptom, the classic combination of three symptoms: headache, fever, and focal neurological deficit occurs in only 20% of cases [5]. In our case, fever was absent, and our patient had mostly seizures and focal neurological deficit. MRI showed two ring-shaped lesions. Nevertheless, the absence of signs of systemic infection and a clear etiology made the diagnosis complicated. This highlights the necessity of evaluating PAVMs as a potential underlying cause of brain abscess in young adults with atypical presentations.

Pulmonary arteriovenous malformations provide a right to left shunt through which venous blood bypasses the pulmonary capillary bed that filters any microorganisms and embolic materials. Therefore, septic emboli and commensal microflora from the mouth can get into the cerebral circulation system and lead to brain abscesses. In this particular case, the causative agent was found to be the organism called Streptococcus milleri belonging to the Streptococcus anginosus group of pathogens.

Antimicrobial therapy was adjusted throughout the patient’s course in consultation with infectious disease specialists. Although current guidelines generally recommend empiric anaerobic coverage for cryptogenic brain abscesses, metronidazole was not included initially because no dental, otogenic, or sinonasal source of infection was identified clinically or radiologically. Following surgical drainage and culture results, therapy was narrowed to trimethoprim/sulfamethoxazole because of its favorable central nervous system penetration. Treatment was later escalated to ertapenem following clinical deterioration and concern for persistent intracranial infection.

While polycythemia was present, current evidence indicates that loss of pulmonary filtration, rather than hypoxia related hematologic changes, is the primary mechanism of abscess formation in PAVM. Other proposed mechanisms, such as microinfarctions acting as infectious niduses, remain theoretical and should be interpreted cautiously [6, 7].

The diagnostic features in our patient are similar to previous cases of PAVM-related brain abscesses, where oral organisms were typically isolated [8, 9]. Although repeated or advanced neuroimaging is important when initial studies are nondiagnostic, in this case, the MRI obtained at presentation was already definitive.

Early surgical evacuation was pursued, consistent with literature suggesting lower rates of treatment failure when surgical intervention is performed in appropriately selected cases, particularly within the first week [10]. Because the PAVM was determined to be the underlying source, angioembolization was performed, improving the patient’s oxygenation and lowering the risk of recurrence.

The negative bubble echocardiogram was surprising given the PAVM later confirmed on CT pulmonary angiography. In this case, standard agitated saline was used and showed a Grade 0 right-to-left shunt. While contrast echocardiography is highly sensitive for detecting pulmonary right-to-left shunts, false-negative results have been reported. Possible explanations include technical factors such as an inadequate Valsalva maneuver, suboptimal contrast opacification, delayed bubble transit beyond the imaging window, or intermittent shunt flow. As detailed procedural documentation was not available for retrospective review, these explanations remain speculative and cannot be confirmed in this case This case emphasizes the value of CT pulmonary angiography when suspicion for PAVM remains high despite a negative initial screening study.

Once the diagnosis of Hereditary Hemorrhagic Telangiectasia (HHT) was made with the Curaçao criteria [11], the management was directed based on second international recommendations, such as screening first-degree relatives, periodic imaging for AVMs, prescribing prophylactic antibiotics for procedures with bacteremia, and counseling in terms of risks related to pregnancy [12]. In this scenario, screening of family members was advised because there was a history of recurrent epistaxis in maternal relatives, though genetic evaluation was limited by cost. Counselling included precautions to prevent air embolism during IV access and the importance of long term follow up.

This case demonstrates the diagnostic challenges and clinical significance of identifying PAVMs in young patients who present with a brain abscess without an identifiable primary source. It shows the importance of heightened clinical awareness of PAVMs as a potential etiology. One of the limitations of this report is that a full evaluation for HHT, including genetic screening, could not be carried out for financial reasons. Furthermore, MRI at follow up was not possible; thus, radiographic resolution of the brain abscess under conservative treatment could not be established.

## Conclusion

This case illustrates a diagnostic challenge where a brain abscess was the initial presentation of an underlying pulmonary arteriovenous malformation (PAVM). Brain abscess in young patients without conventional risk factors should prompt evaluation for PAVMs. This case emphazises the diagnostic challenge of identifying hereditary hemorrhagic telangiectasia (HHT) when the first manifestation is neurological rather than mucocutaneous. Early recognition and management of PAVMs, including angioembolization, are essential to prevent recurrence and better outcomes.

## Supplementary Information

Supplementary Material 1.

## Data Availability

All data generated or analyzed during this case report are included in this article. No additional datasets were generated. Further inquiries can be directed to the corresponding author, subject to ethical and patient confidentiality considerations.
