# Supplementary material for: Previously undiagnosed pulmonary arteriovenous malformation in hereditary hemorrhagic telangiectasia presenting as a brain abscess: a case report
Source: BMC Neurol. 2026 Aug 11;26:707. doi: 10.1186/s12883-026-05220-w (PMC13741449; doi:10.1186/s12883-026-05220-w)
Supplement: Supplementary file 1 [file 12883_2026_5220_MOESM1_ESM.pdf]

CARE Checklist of information to include when writing a case report

CARE case report guidelines

(cc) BY-NC-ND

Topic	Item	Checklist item description	Reported on Line
Title	1	The diagnosis or intervention of primary focus followed by the words "case report"	3,4
Key Words	2	2 to 5 key words that identify diagnoses or interventions in this case report, including "case report"	19
Abstract (no references)	3a	Introduction: What is unique about this case and what does it add to the scientific literature?	43-45
	3b	Main symptoms and/or important clinical findings	47-53
	3c	The main diagnoses, therapeutic interventions, and outcomes	47-53
	3d	Conclusion—What is the main "take-away" lesson(s) from this case?	55-57
Introduction	4	One or two paragraphs summarizing why this case is unique (**may include references**)	83-94
Patient Information	5a	De-identified patient specific information.	96
	5b	Primary concerns and symptoms of the patient.	97-99
	5c	Medical, family, and psycho-social history including relevant genetic information	100-102,
	5d	Relevant past interventions with outcomes	N/A
Clinical Findings	6	Describe significant physical examination (PE) and important clinical findings.	103-105
Timeline	7	Historical and current information from this episode of care organized as a timeline	96-163
Diagnostic Assessment	8a	Diagnostic testing (such as PE, laboratory testing, imaging, surveys).	107, 114,139,148,
	8b	Diagnostic challenges (such as access to testing, financial, or cultural)	207-211
	8c	Diagnosis (including other diagnoses considered)	130-138
	8d	Prognosis (such as staging in oncology) where applicable	N/A
Therapeutic Intervention	9a	Types of therapeutic intervention (such as pharmacologic, surgical, preventive, self-care)	130,135,147,159
	9b	Administration of therapeutic intervention (such as dosage, strength, duration)	130,145,161
	9c	Changes in therapeutic intervention (with rationale)	145,157
Follow-up and Outcomes	10a	Clinician and patient-assessed outcomes (if available)	161-163,204-211
	10b	Important follow-up diagnostic and other test results	N/A
	10c	Intervention adherence and tolerability (How was this assessed?)	to be assessed
	10d	Adverse and unanticipated events	N/A
Discussion	11a	A scientific discussion of the strengths AND limitations associated with this case report	212-216
	11b	Discussion of the relevant medical literature **with references**.	181-188
	11c	The scientific rationale for any conclusions (including assessment of possible causes)	226-233
	11d	The primary "take-away" lessons of this case report (without references) in a one paragraph conclusion	218-224
Patient Perspective	12	The patient should share their perspective in one to two paragraphs on the treatment(s) they received	patient did not comment
Informed Consent	13	Did the patient give informed consent? Please provide if requested	Yes ☑ No ☐
